# Yes- mind the gap!

**DOI:** 10.1186/s12871-021-01250-8

**Published:** 2021-02-09

**Authors:** Uwe Hamsen, Niklas Drotleff, Rolf Lefering, Julius Gerstmeyer, Thomas Armin Schildhauer, Christian Waydhas, TraumaRegister DGU

**Affiliations:** 1 Department of General and Trauma Surgery, BG University Hospital Bergmannsheil, Buerkle de la Camp Platz 1, 44789 Bochum, Germany; 2 Institute for Research in Operative Medicine (IFOM), University Witten-Herdecke, Ostheimer Str. 200, 51109 Cologne, Germany; 3 Medical Faculty University Duisburg-Essen, Essen, Germany

## Abstract

We totally agree with Deana and Colleagues that missing intermediate care 1) might be an explanation for unexpected unfavorable outcome and 2) strengthening of intermediate care has the potential to lower this high rate of unfavorable outcome after ICU discharge. Yes- mind the gap!

## Main text

We want to thank Deana and Colleagues for their interesting comment on our study [1].

The authors discussed our study and they point out a very important issue: patients after critical illness might benefit from a “soft transition” to an Intermediate Care Unit (IMCU) rather than a normal ward. They explain very conclusively the importance to allocate patients to the resources they need regarding, nursing, monitoring, physicians and therapists. Unfortunately our study could not give any information on intermediate care unit usage, as this information is not part of the trauma registry. We ourselves already tried to investigate the value of IMCU in another setting, a large german intensive care registry [2], and we totally agree with Deana and Colleagues that missing intermediate care 1) might be an explanation for unexpected unfavorable outcome and 2) strengthening of intermediate care has the potential to lower this high rate of unfavorable outcome after ICU discharge. Yes- mind the gap!

## Data Availability

Not applicable.

## Abbreviations

ICU: Intensive care unit
IMCU: Intermediate care unit
